# Footprints in Action: How UVA Is Managing Its Sustainability Stewardship

**DOI:** 10.1089/scc.2022.0067

**Published:** 2023-02-23

**Authors:** Elizabeth Dukes, Selina Cheng, Samuel Mogen, James Galloway, Allison Leach, Andrea Ruedy Trimble, Andrew Pettit, Jana Compton, Michael Pennino

**Affiliations:** ^1^ Department of Environmental Sciences, University of Virginia, Charlottesville, Virginia, USA.; ^2^ Sustainability Institute, University of New Hampshire, Durham, New Hampshire, USA.; ^3^ Office for Sustainability, University of Virginia, Charlottesville, Virginia, USA.; ^4^ Environmental Protection Agencies, Office of Research and Development, Center for Public Health and Environmental Assessment Division, Corvallis, Oregon, USA.; ^5^ Environmental Protection Agencies Office of Research and Development, Center for Public Health and Environmental Assessment Division, Washington, DC, USA.

**Keywords:** damage costs, footprints, greenhouse gas, higher education, nitrogen

## Abstract

Evaluating sustainability stewardship at higher educational institutions is essential to working towards improving our environment. Many institutions have used environmental footprint indicators as a way to evaluate, track, and improve their impact on the environment. In this article, we present the web-based Integrated Environmental Footprint Tool (IEFT), which allows users to test how changes in certain activities impact nitrogen (N), greenhouse gases (GHG), phosphorus (P), and water (W) footprints for a university campus. This study uses the University of Virginia (UVA) as a model to show the impacts of their existing sustainability plans on multiple footprint indicators. Strategies from the University of Virginia's (UVA) two exisiting action plans, the GHG Action Plan and the N Action Plan, are evaluated to determine their impact on each of the footprints (GHG, N, P, and W). Based on the 2025 goal year, the strategies in these action plans are estimated to reduce the GHG, N, P, and W footprints by −38%, 32%, 25%, and 2.7% respectively. The damage costs associated with GHG and N footprints are assessed and reveal a 38 percent reduction in damage costs for GHG and a 42 percent reduction in costs for N. Using the IEFT to evaluate the impact of these action plan strategies, UVA optimized environmental outcomes. The model shown here can be used at other institutions to evaluate the environmental impact of planned changes to an institutions' operations.

## Introduction

### ***Environmental Footprint Indicator*** *s*

Tracking environmental impact at any scale can be challenging. Footprint models attempt to address this challenge by providing a quantitative way to show an entity's pressure on resource use. These include resources used for energy generation and food production and consumption. These footprint indicators can allow individuals (D'Odorico et al., 2019; Gao et al., 2014 Leach et al. 2012, 2016), institutions (Castner et al. 2017; Leach et al. 2017; Valls-Val & Bovea, 2021), and communities (Dukes et al. 2020; Shan et al., 2017; Stanganelli, 2020; Wiedmann & Allen, 2021) to make informed decisions on reforming practices and policies to improve environmental outcomes.

Estimating multiple indicators give institutions a broader understanding of their environmental impacts and what is needed to reduce those impacts. Focusing on one indicator while ignoring the impacts of another could lead to trade-offs that create unintended environmental consequences (i.e., “pollution swapping”). Several entities have taken the approach of looking at multiple indicators (e.g., nutrients, biotic integrity) to assess environmental quality. These indicators have been used within watersheds (Flotemersch et al., 2015), protected areas (Cook et al., 2012) and endangered ecosystems (Mouillot et al., 2017), and even the entire earth (Sherwood et al., 2020). This study assesses the impact of different management strategies on greenhouse gas (GHG), nitrogen (N), water (W), and phosphorus (P) footprints.

### Quantifying Environmental Footprints as Damage Costs

In order to quantify some of the impacts that environmental change has on human health, researchers have assigned dollar values to greenhouse gas (Tol, 2005) and reactive nitrogen emissions (Birch et al., 2011). These can be used to estimate damage costs associated with GHG and N footprints (Compton et al., 2017). Damage costs associate dollar values to degradation of environmental or human health resulting from anthropogenic emissions. Damage costs for GHG and N emissions include the costs for human respiratory health, treatment of public drinking water, and ozone damage to crops. These assessments can provide a common metric for comparing impacts of different activities, and to evaluate the connections between equity and the environment. Cost assessments could be used to inform decision making around environmental dilemmas.

### Educational Institution Footprints

Higher education institutions have been a place of innovation in developing and using footprint indicators. Institutions have signed their own climate commitments or made commitments via third-party organizations such as Second Nature to reduce their greenhouse gas emissions (Second Nature, 2018). More than 400 institutions have signed on to a President's Climate Leadership Commitment, using tools like the Sustainability Indicator Management and Analysis Platform (SIMAP). Others have created their own tools and developed their own environmental footprint approaches (Clabeaux et al., 2020).

Though footprints are a common way to estimate institutional environmental impacts, footprint reporting is relatively new for colleges and universities. Although the most commonly tracked and reported environmental footprint in higher education is the GHG footprint, dozens of institutions are now estimating their nitrogen footprint (Castner et al., 2017). Although many institutions track their direct water usage, tracking the virtual water footprint is relatively new (Natyzak et al., 2017). The phosphorus footprint is entirely new and not yet tracked by institutions (Metson et al., 2020).

### Defining Environmental Footprint Indicators

A GHG footprint is defined as the total amount of carbon dioxide, methane, and nitrous oxide measured in carbon dioxide equivalents, produced as a result of an entities function. GHG footprints are often synonymous with carbon (C) footprints. To be consistent with terminology used in UVA's action plans, the term GHG is used throughout this work. GHG emissions contribute to global climate change. A major source of GHG emissions at an institution level is fossil fuel combustion (e.g., stationary fuels, transportation, purchase electricity) (Leach et al., 2017). Tracking the GHG footprint was spurred by the American College and University Presidents' Climate Commitment, Presidents Climate Leadership Commitment, 2023 a 2006 commitment signed by many higher education institutions that have agreed to reduce their GHG emissions (Cleaves et al., 2009; Sharp 2002). This has now evolved to the Second Nature Presidents' Climate Leadership Commitments. Reporting GHG emissions has helped campuses track their progress toward carbon neutrality goals over time, and it has also brought awareness to climate change issues through education, research, and outreach.

Institutions have also studied N footprints as part of the Nitrogen Footprint Tool Network (Castner et al., 2017). The N footprint is a measure of the amount of reactive nitrogen released to the environment as a result of an institution's resources use such as food, energy, and transportation. Reactive nitrogen is essential for food production but too much reactive nitrogen can cause a cascade of negative environmental and human health impacts, ranging from smog to eutrophication (Galloway et al., 2003). The food portion is often 50 percent or more of an institution's N footprint, often driven by meat (Castner et al., 2017). Using the N footprint as a measure of sustainability adds to GHG analysis, especially by providing a metric-based analysis of food sustainability (Castner et al., 2017; Dukes et al., 2021; Leach et al., 2012).

Direct water use is often associated with chiller plants and use within dining halls, labs, educational buildings, and dormitories. While it is important for an institution to measure, it does not capture water used upstream. Virtual water use is the measure of water incorporated in the production and manufacturing of goods such as food and electricity (Natyzak et al., 2017). Using this metric expands the view of impacts of water use from a local perspective to a more global perspective of water insecurity.

Phosphorus (P) footprint is a more recently developed metric used at the global scale. The P footprint is a measure of the amount of P lost to the environment as a result of an institution's resource use such as food and wastewater. Similar to N, P is necessary for life and is often used in fertilizer for soils (Metson et al., 2020). However, too much P fertilization causes eutrophication and degradation of oceanic and freshwater systems. Unlike N, P cannot be manufactured. Increased cycling in P can a threat to food security as less P is available for crops and more P is added to aquatic systems causing eutrophication (Metson et al., 2020). Food production, fertilizer use, and wastewater are important sectors that contribute to an entity's P footprint. Tracking a P footprint at a university can provide information on how to shift demand for P.

The mission of higher education institutions is to educate and to conduct research, which is one of many reasons institutions are the innovators in this field. Using institutions as living labs for environmental footprint tracking and reduction can provide examples to surrounding communities (Dukes et al., 2021). Other benefits to working at the institutional level are having a clearer boundary to work within when calculating environmental footprints and having a well-defined decision-making board.

### Footprint Tracking and Goal Setting at the University of Virginia

Starting in 2009, the University of Virginia began tracking environmental footprints with the calculation of UVA's greenhouse gas (GHG) footprint (metric tons of CO_2_ equivalent or MTCDE) using the Clean Air Cool Planet (CACP) calculator. In 2017, the CACP calculator moved to an online platform called the Sustainable Indicators Management and Analysis Platform (SIMAP) combining nitrogen (N) and GHG footprints where UVA currently tracks both its GHG and N footprints. In 2011, UVA proposed to reduce the GHG footprint by 25 percent by 2025 compared to 2009 levels. This goal was reached six years early, with a 27 percent reduction in GHG emissions by 2019 (University of Virginia, 2019c).

UVA began tracking its N footprint with the development of the nitrogen footprint tool (NFT) for institutions (Leach et al., 2013). The first calculation was completed for the baseline year of 2010. In 2013, UVA set an N footprint reduction goal of 25 percent by 2025 (UVA Nitrogen Action Plan, 2019).

The W footprint of UVA was calculated as a research activity in 2015 (Natyzak et al., 2017). This was the first evaluation of indirect water use at the university level. In 2019, UVA set a goal to reduce direct water use by 30 percent by 2030 based on 2010 levels.

The P calculation presented in this article is the first to track the phosphorus footprint at UVA. There are currently no plans to reduce the P footprint in UVA's strategic plans.

In December 2019, UVA's Board of Visitors passed a resolution on new sustainability goals for UVA, which increased environmental footprint reductions and accelerated timelines. UVA's GHG goal is now to be carbon neutral by 2030 and fossil fuel free by 2050. UVA also committed to reduce water and reactive nitrogen losses to the environment by 30 percent, reduce its waste footprint to 30 percent (relative to 2010 baselines), and increase sustainable food purchases by 30 percent, all by 2030 (A Great and Good University, 2019).

### Study Objectives

Given the goals at UVA, the objectives of this study are to: 1.) present UVA's 2016 and 2025 projected business as usual (BAU) GHG, N, P, and W footprints using the web-based Integrated Environmental Footprint Tool (IEFT); 2.) present the UVA 2019 GHG Action Plan (GHGAP) and 2019 Nitrogen Action Plan (NAP); 3.) run scenarios in the UVA action plans in the IEFT and examine the synergies and tradeoffs between the overall impacts on the GHG, N, P, and W footprints; and 4.) determine damage costs associated with UVA's GHG and N footprints using the IEFT.

## Methods

### Integrated Environmental Footprint Tool

A web-based application, called the Integrated Environmental Footprint Tool (IEFT) was created to see how the GHG, N, P, and W footprints and GHG and N damage costs at UVA change when different behaviors and campus activities are changed (C, N, P, & W Footprints (UVA 2016 Data), n.d.). The application was created using R, which is a programing language.

The GHG represents the total GHG emissions from campus activities, reported in units of carbon dioxide equivalents (Table 1). The major sources are fossil fuel combustion (e.g., stationary fuels, transportation, purchased electricity), food production, refrigerants, animals, and fertilizer application. The GHG footprint is calculated by multiplying the activity data (e.g., kWh electricity purchased, tons of coal used, automobile miles driven, pounds of fertilizer applied) by the carbon dioxide, methane, and nitrous oxide emissions factors, as appropriate. All emissions factors were collected from the carbon and nitrogen footprint tool SIMAP (Leach et al., 2017), which compiles US emissions factors for fuel use (e.g., Environmental Protection Agency, Bureau of Transportation Statistics), food production (Heller and Keoleian, 2014), and refrigerants and chemicals (Devotta & Sicars, n.d.).

**Table 1.** The Carbon, Nitrogen, Phosphorus, and Water Footprints: Emissions Types, Normalized Units, Key References, and Sectors Impacted

	Emissions Types	Normalized Units	References	Sectors Impacted
Greenhouse Gas footprint	CO_2_, CH_4_, N_2_O, and refrigerants	Metric tons CO_2_ equivalents	Leach et al. (2017), University of New Hampshire (n.d.)	Stationary fuels, electricity, transportation, fertilizer, animals, food, refrigerants, and chemicals
Nitrogen footprint	NO_x_, N_2_O, other N	Metric tons of N	Leach et al. (2017), University of New Hampshire (n.d.)	Stationary fuels, electricity, transportation, fertilizer, food, and wastewater
Phosphorus footprint	P	Kg of P	Metson et al. (2020)	Food, fertilizer, and wastewater
Water footprint	Blue water, green water^a^	Gallons of water	Natyzak et al. (2017)	Stationary fuels, electricity, transportation, food, direct water use, and wastewater

^a^ Blue water refers to the water sourced from ground or surface water; green water refers to water from precipitation stored in plants (Hoekstra et al., 2012).

The nitrogen footprint is the total reactive N released to the environment from campus activities, reported in units of metric tons of N (Table 1). The major sources are food production, fossil fuel combustion, wastewater, and fertilizer application. The N footprint is calculated by multiplying activity data (the same ones used for other footprints) by the appropriate emissions factor (NO_x_, N_2_O, and other N). All N emissions factors were collected from SIMAP (Leach et al., 2017).

The phosphorus footprint is the total P released to the environment from campus activities, reported in units of kilograms of P (Table 1). The major sectors for the P footprint are food production, wastewater, and fertilizer application. P emissions from fossil fuel combustion are negligible and were therefore excluded from this analysis. All emissions factors were collected from Metson et al. (2020), which presents the P footprint of the United States.

The water footprint is the total freshwater usage, reported in cubic meters of freshwater consumed (Table 1). The water footprint can be reported as the blue water footprint (irrigation), the green water footprint (precipitation), and the grey water footprint (amount needed for dilution of pollutants to safe levels). This article reports the blue and green water footprints for energy use (e.g., hydropower) and food production. The footprint also includes direct water use by a campus. All emissions factors were collected from Natyzak et al. (2017), which presents the water footprint for UVA.

The methods, system bounds, and data sets in the IEFT application were based on SIMAP, a web-based tool for calculating campus carbon and N footprints (Leach et al., 2017; University of New Hampshire, n.d.). Emissions factors were added for P and W and applied to the same campus activity data so that all four footprints could be directly compared. More information and equations detailing the calculation of each footprint can be found in the Supplementary Material.

The baseline GHG, N, P, and W footprints were calculated based on 2016 usage data at UVA for the following categories: purchased electricity, food, on-campus stationary sources, direct transportation sources, commuting, fertilizer, animals, refrigerants and chemicals, wastewater, and direct water use (see Supplementary Material Table S1).

**Figure 1.**
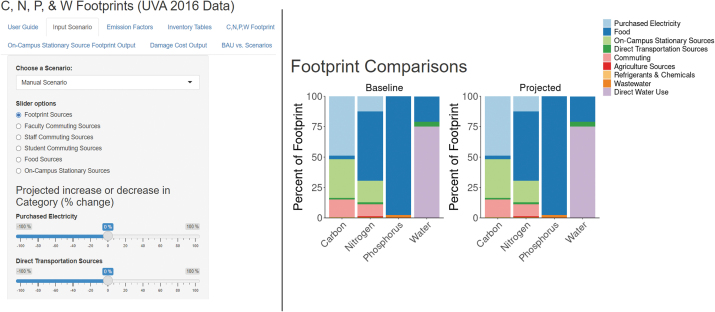
Interface of the IEFT tool where scenarios can be chosen or created (left) and one of several images of results (right). The user interface allows for easy access to both project growth and run reduction scenarios (C, N, P, & W Footprints (UVA 2016 Data), n.d).

The IEFT allows users to evaluate the impact of a projected change in resource use on the four footprints and damage costs associated with the GHG and N footprints. The IEFT application displays tables and figures to compare the baseline footprint with projected scenarios. Within the IEFT application, there is the ability to change emissions factors and view impacts of strategies on specific categories. These features allow users to model the impacts scenarios will have on their institution's footprints (See Figure 1).

### Data Gathered and Used for IEFT Calculations

All data used in the IEFT were gathered by either UVA Facilities Management, Dining, or Health System operations. The data were entered into the IEFT for calendar year 2016 to calculate the GHG, N, P, and W footprints simultaneously (see Supplementary Table S1).

In order to achieve these reduction goals, UVA has written two relevant action plans outlining strategies to reduce these footprints: the Greenhouse Gas Action Plan (2017) and the Nitrogen Action Plan (2019) (Table 2). The aim of these action plans is to outline strategies the university will implement by 2025 in order to meet environmental sustainability goals. All strategies evaluated in this study were derived from these action plans. The column on the left describe the strategies listed while the two columns on the right indicate whether or not the strategy is included in each plan: Y (yes, it is included) or N (no, it is not included). More information on individual scenario improvements can be found in Supplementary Table S2.

**Table 2.** List of Strategies in the University of Virginia Sustainability Action Plans

	Strategy	Included in N Action Plan	Included in GHG Action Plan
1	Fuel optimization	Y	Y
2	Chilled water plant efficiency improvements	Y	Y
3	Heat plant efficiency improvements	Y	Y
4	Distribution efficiency/low temperature/hot water (LTHW)	Y	Y
5	Dominion electricity grid improvements	Y	Y
6	On-grounds solar	Y	Y
7	Off-grounds utility scale solar (UVA Hollyfield Solar facility)	Y	Y
8	Off-grounds utility scale solar (UVA Puller Solar facility)	Y	Y
9	Additional off-grounds utility scale solar	Y	Y
10	Existing building improvements	Y	Y
11	Green Building Standards	Y	Y
12	Green Labs	Y	Y
13	Gasoline fleet improvements	Y	Y
14	Diesel fleet improvements	Y	Y
15	Outreach and Engagement	Y	Y
16	Green IT	Y	Y
17	15% vegetarian meal replacement	Y	N
18	Meat-focused café becomes plant forward	Y	N
19	Plant forward Castle	Y	N
20	Plant forward theme-meals (3 per month)	Y	N
21	Plant forward Fridays in residential dining halls	Y	N
22	20% beef replaced with chicken	Y	N
23	Blended burgers in dining halls	Y	N
24	10% health system beef replaced with chicken	Y	N
25	15% burgers replaced with Beyond burgers	Y	N
26	Mindful Mondays	Y	N
27	Beef-free station for 6 months	Y	N
28	50% avoidable food waste reduced	Y	N
29	50% food is sustainable	Y	N

### Estimating Business as Usual and Projection N Footprints

The default values in the IEFT are based on UVA's 2016 operations. To evaluate the impacts of the Nitrogen and Greenhouse Gas Action Plans, UVA's activity plan was projected to the goal year of 2025 based on a business-as-usual (BAU) 2016 scenario. These estimates were made based on population, gross square footage, and food purchasing trends (see Supplementary Table S8) and were entered into the IEFT. We calculated UVA's 2025 baseline activities to reflect a business-as-usual (BAU) scenario based on the projected increase in gross square footage, population, and food demand from 2016 to 2025. These changes resulted in increases across all four footprint indicators from 2016 to 2025. The GHG footprint increased by 19%, N footprint by 18%, P footprint by 18%, and W footprint by 4% from 2016 to 2025, predominantly due to the food and energy sectors (Table 2). For each scenario, the BAU calucated within the IEFT was altered by sector, depending on the specific strategy (see Supplementary Table S1, Table S2, Table S3, Table S4, and Table S5).

### Action Plan Strategy Analysis

Each scenario listed in Table 2 was evaluated individually within the framework of the IEFT. The scenarions were combined to determine the overall impact of the two action plans on the four footprints (GHG, N, P, and W) and two damage costs (GHG and N). The calculations from three of the most impactful action plan scenarios are highlighted, two relating to food and one relating to energy. These strategies were: 20 percent beef to chicken replacement (Table 2, #22), 15 percent vegetarian meal replacement (Table 2, #17), fuel optimization (Table 2, #1). The final scenario run included all strategies from both action plans (Table 2). Calculations for all scenarios can be found in the Supplementary Material.

The IEFT data and the default values represent the 2016 baseline food, energy, and transportation consumption at UVA. The percent reductions required by the action plan strategies are based on a 2025 BAU baseline to account for population increases.

### Calculation of Damage Costs Due to GHG and N Release

Damage costs associated with both GHG and N were calculated for different usage categories (electricity, food, commuting, etc.). For N only, damage costs were also calculated for social-environmental sectors (human health, agriculture, ecosystems, climate), various media (land, water, air), and different N types (NO_3_, NH_3_, N_2_O, dissolved N). Damage costs associated with specific N fluxes were measured in terms of dollar per kg N released to the environment. The specific damage costs were largely obtained from a regional study of the Chesapeake Bay watershed (Birch et al., 2011), in which UVA is located, supplemented with other values from the literature (Compton et al., 2017). The values represent incremental or marginal increases in cost associated with the impacts of N use on a per unit of N basis and assume a linear response function (see Supplementary Table S9).

For the social costs of GHG (CO_2_-C and N_2_O-N), there is significant variance in the estimates depending on scope (global vs. national impacts) and discount rates. In a literature review, Tol (2005) found modal estimates of only $2/t GHG and concludes that mean values are likely less than $50/t GHG. The Interagency Working Group on the Social Cost of Greenhouse Gases (2016) estimated a global-scale social cost of GHG of from $14 to $74 per metric ton of carbon dioxide equivalent (MTCDE) for a range of discount rates, while current estimates from the EPA for national costs are from $1 to $7 per MTCDE. Values of $30 per ton of CO_2_ equivalent were used as an intermediate value. All dollar values were adjusted for inflation and pegged to the US dollar in 2016 using the consumer price index (US Bureau of Labor Statistics, n.d.), to coincide with the 2016 IEFT data.

Marginal damage costs associated with GHG and N released by UVA were calculated using the IEFT based on GHG and N release by chemical compound and mode of impact. The N release from NO_x_-N, NH_3_-N, and N_2_O-N were combined with the damage costs associated with those forms. Virtual N from food production was apportioned into dissolved N, NH_3_, NO_x_, and N_2_O (Houlton et al., 2013). The hydrologic N released to water was used to estimate the damages associated with release to surface water, groundwater, and coastal systems.

Currently, there are no damage cost evaluations completed for P and W. If these data become available in the future, they can be included damage cost estimations.

### Evaluating the Damage Cost of the Fuel Optimization Scenario

Changes in energy usage from a 2016 baseline scaled to 2025 were estimated, and changes in NO_x_ emissions were subsequently determined using emissions factors for each fuel type. Economic benefits of changes in NO_x_ emissions were calculated using health and economic damage estimates in the Chesapeake Bay Watershed for precursors of ozone and particulate matter (PM_2.5_) (Birch et al., 2011). For each ton of NO_x_ reduction, a certain amount of damage would be mitigated via reductions in formation of harmful ozone and PM_2.5_ formation as calculated using Equation 1.

Monetary Savings, NO_x_=(NO_x_ Emissions_BAU_ – NO_x_ Emissions_2025NGONLY_)×$ damage per ton NO_x_

## Results

### Action Plan Strategy Results

**Figure 2.**
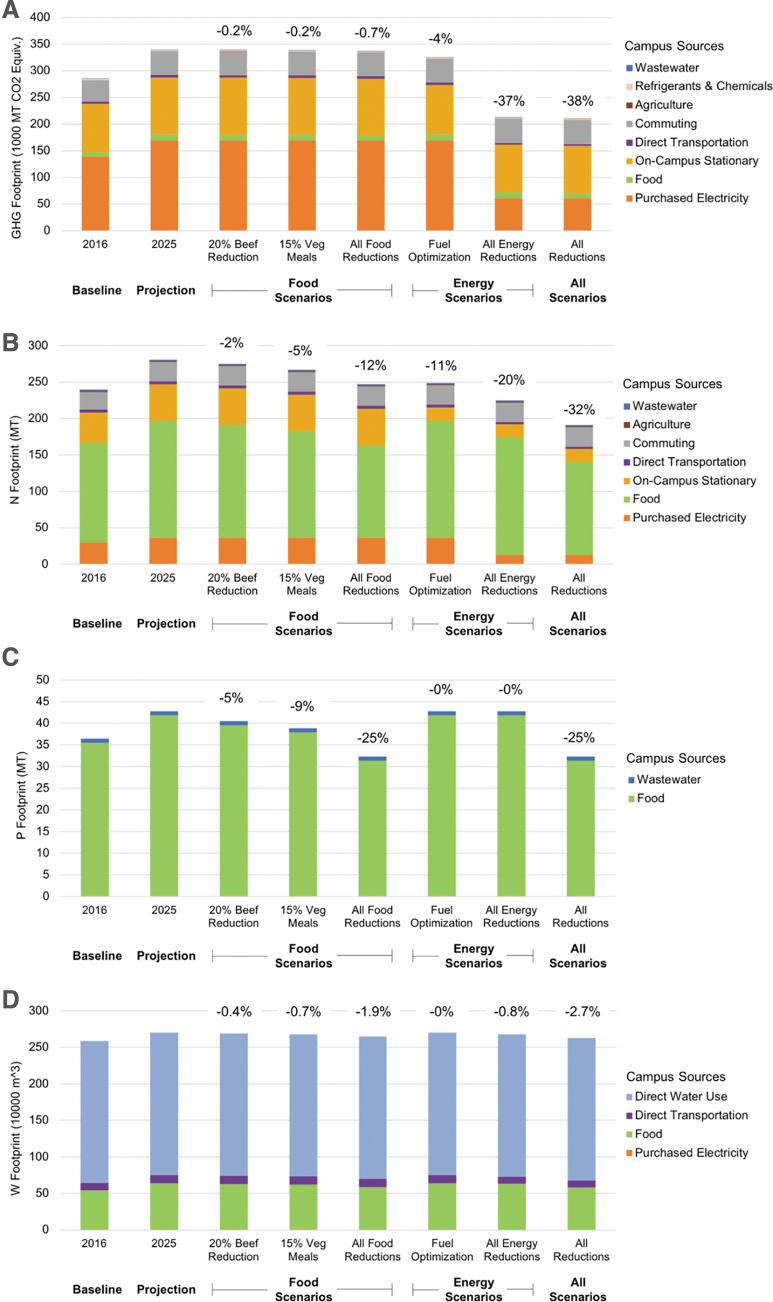
Results for the GHG (a), N (b), P (c), and W (d) footprints for UVA. The 2016 baseline year and 2025 business-as-usual projection year are shown as the first two bars on the horizontal axis of each panel. Bars 3-8 are reduction strategies applied to the 2025 business-as-usual numbers. In order, these bars show a 20% beef replacement with chicken by weight (Table 2, #22), a transition to 15% vegetarian meals in all dining halls (Table 2, #17), all food reductions (Table 2, #17-29), fuel optimization (Table 2, #1), all energy reductions (Table 2, #1-16), and all reduction scenarios combined (Table 2, #1-29). Percentages listed over the figures correspond to percent reductions in footprints from 2025 business-as-usual to 2025 with the given scenario. Consumption categories that did not impact certain footprints were omitted from that footprint's graph.

The 2025 BAU scenario indicates UVA would not meet its goals of a 25% N and GHG footprint reduction by 2025 unless further actions were taken. The UVA action plan scenarios were then implemented by entering the appropriate data in to the IEFT tool (Table 2). Many of these changes resulted in a decrease from 2025 BAU values and a decrease from 2016 values. The N footprint was reduced by 20% and GHG was reduced by 26%. When all scenarios were implemented, the overall changes from the GHG, N, P, and W footprints were: -38%, -32%, -25%, and -2.7%, respectively. Strategies focusing on the energy and food consumption categories were also run separately to demonstrate the large impacts that these sectors have on the footprints. The strategy for 20 percent beef replacement with chicken resulted in a 0.2 percent reduction in GHG, 2 percent reduction in N, 5 percent reduction in P, and 0.4 percent reduction in the W footprint. The fuel optimization strategy resulted in footprint reductions of 4 percent for GHG, 11 percent for N, 0 percent for P, and 0 percent for W (Figure 2). Exact values for each category outputs can be found in the Appendix (Supplementary tables S6, S7, and S8).

### Impactful Food Scenarios

The two food scenarios with the largest impact on all footprints were chosen for further analysis. These scenarios were a 15 percent vegetarian meal replacement scenario (Table 2, #17) and a replacement of 20 percent of beef with chicken (Table 2, #22). These scenarios were shown individually in each footprint in Figure 2, A-D.

**Figure 3.**
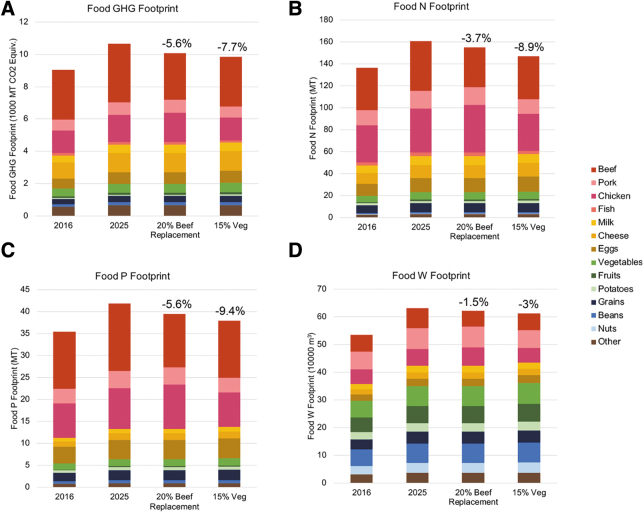
The food GHG footprint (A), nitrogen footprint (B), phosphorous footprint (C), and water footprint (D), for each of the 4 scenarios.

The scenarios were also further evaluated to show their impact on the food footprints, specifically (Figure 3). For each food scenario, the total weight of food was kept the same to ensure the number of meals stayed consistent across scenarios while the content changed. The 20 percent beef to chicken scenario resulted in reductions in the food sector: 5.6% for GHG, 3.7% for N, 5.6% for P, and 1.5% for W, compared to BAU. The 15 percent vegetarian meal replacement resulted in reductions in the food sector: 7.7% for GHG, 8.9% for N, 9.4% for P, and 3.0% for W. Neither scenario altered the total weight of food consumed.

### Impactful Energy Scenarios

**Figure 4.**
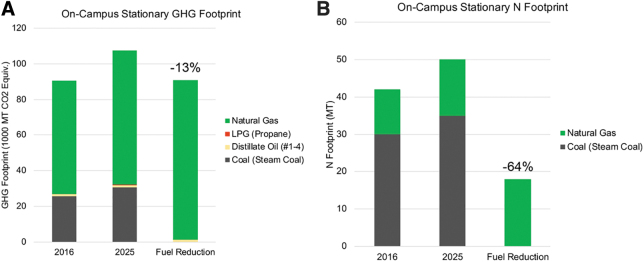
The GHG (A) and N (A) footprints of the on-campus stationary footprint split into fuel type in 2016, 2025 business-as-usual, and 2025 with the fuel optimization scenario (Table 2, #1).

Similar to the two food scenarios chosen for additional evaluation, the fuel optimization scenario (Table 2, #1) was chosen for additional analysis of the energy sector, specifically the on-campus stationary category. This scenario switched fuel use from coal to natural gas for the UVA on-site heating plant. This scenario only affected the GHG and N footprints (see Figure 4). The fuel reduction scenario resulted in a 13 percent reduction in the GHG footprint and a 64 percent reduction in the N footprint.

### Damage Costs

**Figure 5.**
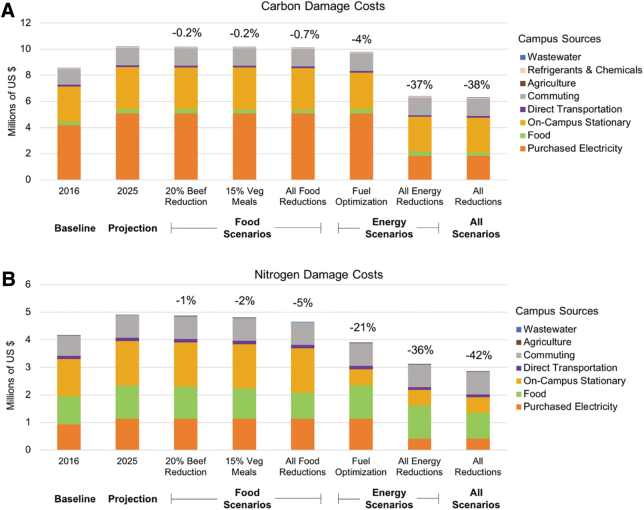
Estimated damage costs by source activity for GHG (a) and N (b). The estimated percent damage cost reductions are shown above each bar for the 2016 baseline, 2025 business-as-usual projection, individual food scenarios, individual energy scenarios and all of scenarios combined. These damage costs are shown by sector (wastewater, agricultural, commuting, direct transportation, on-campus stationary, food, and purchased electricity).

Next, the total GHG and N damage costs were assessed for the original 2016 data, the 2025 BAU scenario, and scenarios in the NAP and GHGAP. Additional figures regarding damage costs can be found in the Appendix (see Supplementary Material, Table S8). When all action plan scenarios were implemented, GHG damage costs were reduced by 38 percent and N damage costs were reduced by 42 percent. Implementing only energy reduction scenarios resulted in a 37 percent reduction in GHG damage costs and a 36 percent reduction in N damage costs (Figure 5), whereas implementing only food reduction scenarios led to a 0.7 percent reduction in GHG damage costs and a 5 percent reduction in N damage costs (Figure 5).

**Figure 6.**
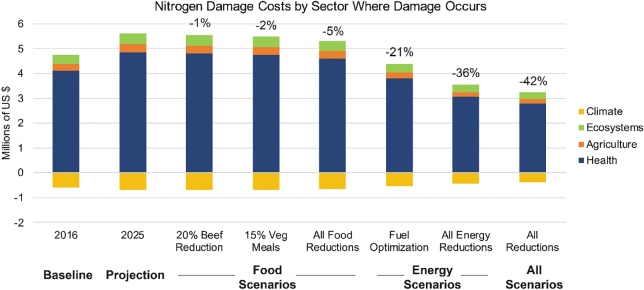
Estimated damage costs associated with N by impacted sector. The estimated percent damage cost reductions are shown above each bar for the 2016 baseline, 2025 projection year, individual food scenarios, individual energy scenarios and all of scenarios combined. These damage costs are shown by category impacted (climate, ecosystems, agriculture, health). Aerosols containing NOx and NHy can produce small, short-lived cooling effects (Mahowald, 2011; Shi et al., 2015) and thus we include the dollar value associated with the cooling effects of aerosol as a benefit (i.e., negative damage cost) under the climate category in this paper, as others have done (Van Grinsven et al., 2013).

Reducing NO_x_ emissions also reduces by-products that are harmful to human health (e.g., ozone, particulate matter). Thus, reducing emissions can have concrete economic benefits due to avoidance of negative health impacts. Implementing the fuel optimization scenario would result in estimated savings of $370,000 due to reductions in ozone precursors and $635,000 due to reductions in emissions of PM_2.5_. This gives total community health cost savings of around $1 million due to the fuel optimization scenario.

## Discussion

### Synergies and Tradeoffs between the GHG, N, P, and W Footprints

In the suite of scenarios run from UVA's 2025 action plans, there were no classic tradeoff scenarios in which one footprint increased as a result of a strategy put into place to decrease another. However, in many cases scenarios have a greater impact on one footprint compared to another. For example, energy scenarios have a relatively large impact on the GHG and N footprints, but an absent or negligible impact on the P and W footprints. In contrast, the food scenarios have a large impact on the N and P footprints, and a relatively small impact on the GHG and W footprints. The energy and food reduction scenarios complement each other in creating reductions across all footprints. This suggests that it would be beneficial to implement reductions across a variety of categories to achieve an impact across all footprints, rather than focusing on just energy or just food consumption (Figure 2).

It is notable that no scenarios have a large impact on the W footprint; the implementation of all reduction scenarios leads to only a 2.7 percent reduction in the W footprint (Figure 2). Most of this reduction can be attributed to the implementation of food scenarios (Figure 3), though reductions in transportation and purchased electricity also have a small impact. Reductions in the water footprint can be best achieved by reducing UVA's direct water use. UVA is currently in the process of finalizing and implementing a Water Action Plan to reduce potable water use by 30 percent below 2010 levels by 2030. Scenarios mentioned in the preliminary Water Action Plan that are not addressed here include building-specific water improvements, water technology improvements (e.g., toilets, washing machines, refrigerators, etc.), and implementation of Green Building Standards for water. If these water reduction scenarios are implemented in conjunction with the food and energy reduction scenarios, UVA will see larger impacts across all footprints.

It is also important to note that only by combining all scenarios is UVA able to reduce any one footprint below 2016 levels due to projected growth of the university in both population and gross square footage. Even though reduction below 2016 levels is only possible when all scenarios are combined, each individual scenario was shown to have a reduction in footprints when compared to BAU growth.

While growth increases the total university footprint, there is also a transfer of footprints happening that is difficult to capture. As the university population and operations grow, there are students and staff previously outside of the scope of the university being included. This reinforces the idea of working to create multifaceted approaches addressing multiple sectors and scopes of the university's operations to move toward reaching reduction goals. As the university grows, there is a need to optimize resource use to reach absolute footprint goals while operating at a larger capacity. The creation of these two action plans addresses each portion of the GHG and N footprints through several important sectors (purchased electricity, on-campus stationary, and food) by including multiple strategies within each. These action plans address both optimizing use by improving fuel efficiency and reducing food waste as well as switching to more sustainable options by moving to renewable fuels and increasing plant based options.

### Impactful Scenarios

Of the many food scenarios explored, the 15 percent vegetarian meal replacement and the 20 percent beef replacement with chicken scenarios are highlighted in this article. These two scenarios turned out to be the most impactful ones and involved the greatest reductions in the various categories of meat consumption. The 15 percent vegetarian meal replacement scenario was the most impactful food scenario as it required a decrease in consumption of all meats and a corresponding increase in all substantive vegetarian alternatives (e.g., beans, eggs, grains, etc.) (Figure 2). The 20 percent beef replacement with chicken scenario did not involve a net reduction in the amount of meat consumed which may be more accepated by customers at the UVA dining halls. The 20 percent beef replacement scenario demonstrates that an appreciable footprint reduction can still be achieved even if overall meat consumption remains unchanged (Figure 3).

The fuel optimization scenario was highlighted as the energy reduction strategy to further analyze for this study. This scenario in particular was chosen because the strategy is an integral piece of the existing GHG and N Action Plans. Switching from coal combustion to natural gas combustion will generate a significant reduction in on-campus stationary combustion emissions (Figure 4). Additionally, this strategy is already in the process of being implemented by UVA, and the estimates calculated by the IEFT can be measured against real-time data in the near future. Lastly, the strategy could have considerable health benefits for the local community, valued at close to $1 million in costs avoided (Figure 5).

### *Integration of Damage Costs for the GHG and N Footprints*

This study combines the social costs of carbon and nitrogen into one analysis. This is a challenging step because there are substantial differences in the impacts of carbon and nitrogen in terms of sources, forms, impacts, and spatial dynamics. Carbon impacts have been estimated by hundreds of papers (Nordhaus, 2017) and are modeled as one evenly-distributed form (CO_2_) whose impacts have one primary driver (change in temperature). Damages are thus assumed to be independent of the origin of the carbon emissions (Keeler et al., 2016). In contrast, there are currently only a handful of estimates of N or P damages, and cost metrics are dependent on complex interactions between the forms released and the impacted systems (Birch et al., 2011; Sobota et al., 2015; van Grinsven et al., 2013). Impacts by sector and type are broken out by type in the Appendix (Supplementary Figures S1 and S2 and Supplementary Table S9) Recognizing these differences in impacts, very different methods to estimate the damages were used.

Both carbon and nitrogen have significant costs associated with their releases to the environment. Including damage costs provides a more complete and more complex picture of the connections between specific environmental releases and their health and environmental impacts. Carbon damage costs were only about twice the nitrogen costs, even though the amounts released were a thousand-fold greater for carbon than for nitrogen. This is likely because damages due to nitrogen can have significant human health impacts, particularly with regard to human respiratory health.

There were also important differences between the drivers of the N impacts and drivers of the GHG-related impacts. Most of the carbon impacts were associated with purchased electricity and on-campus stationary sources while nitrogen damages were largely driven by food consumption (Figures 4 and 5). Including both N and GHG damage costs thus allows UVA to examine the impacts of its consumption across several categories (food and energy) rather than just one. Including the nitrogen damage costs in particular may lead the university community to more carefully consider and examine the impacts of food choices on their footprints.

With UVA's fossil fuel-free goal, quantifying the community impacts and benefits of using less pollution-intensive fuel will be useful in understanding downstream economic savings. Damage cost estimates do not represent the full savings but rather just the avoided costs of the predicted damages. Many of these scenarios will cost money to implement, which is not incorporated here. It is hoped that a more complete picture of social impacts in the future can be accomplished by expanding beyond the Central Grounds heating plant and including impacts from emissions related to electricity generation.

### Future Plans for UVA's Footprints

The IEFT can help determine the broader impact of the scenarios in each action plan. UVA can use this tool to evaluate additional strategies to meet more ambitious environmental sustainability goals before choosing which strategies to add to a new action plan. In an institution of UVA's size with many stakeholders, buy-in is more readily achieved when a strategy is comprehensively evaluated. The IEFT calculates the estimated impact a strategy has toward reducing institutional GHG, N, P, and W footprints. This reduces the need for UVA to invest in developing individual calculations for each environmental factor. Additionally, the tool calculates the estimated impact a strategy may have on all of these factors at once, providing the user with the estimated co-benefits of each strategy. A next step is to explore ways to integrate water and phosphorus into more established institution footprint tools such as SIMAP. This information can be helpful for a decision maker not only to determine which strategy to include in an action plan, but to also determine priority for a strategy to be implemented at UVA and other institutions.

Tracking more environmental footprints introduces the ability to track progress in a campus's local environment. The GHG footprint focuses specifically on the contribution to global climate change, but the N, P, and W footprints all have impacts on local environmental quality. For example, the local N footprint can be tracked separately to specifically improve a campus's local environment. Local N footprint reductions can reduce eutrophication in local waterways and decrease local instances of smog (Dukes et al., 2020; Stanganelli et al., 2020). This provides great potential to connect sustainability research with the surrounding environment and to engage the local community.

Finally, the introduction of damage cost indicators goes beyond direct environmental impacts by connecting a university's activities with human health and livelihood. Damage cost indicators assess the monetary cost associated with environmental damage, such as GHG and N emissions, taking into account factors like the impact of air quality on human health. For example, although NO_x_ emissions make up a small proportion of the total N footprint, NO_x_ emissions incur large damage costs because of their disproportionately harmful effects on human health. Considering the damage cost indicators in campus planning would allow campuses to prioritize reducing aspects of their footprint that cause the most detrimental impacts.

## Summary

This study presents the implementation of the UVA GHG and N Action Plans using the Integrated Environmental Footprint Tool. The IEFT empowers users to evaluate changes in their GHG, N, P, and W footprints in one platform by allowing users to alter their baseline activity data depending on certain reduction strategies. The strategies implemented in this study were taken from UVA's action plans that aim to reduce university GHG and N footprints. Though the action plans were GHG- and N-specific, running some scenarios also revealed synergistic reductions for W and P. After all scenarios in these action plans were implemented, the GHG, N, P, and W footprints changed by -38%, -32%, -25%, and -2.7%, respectively. Each strategy had variable impacts on the different footprints, with some strategies having larger impacts on one footprint than another. Implementing multiple, complementary strategies can thus lead to greater reductions across all footprints.

UVA has set additional reduction goals for GHG, N, and W footprints. Using the IEFT, the university can evaluate how different combinations of strategies can affect all four footprints, allowing UVA to choose the combination that is most effective. Accompanying these footprints with additional information such as health costs savings can also provide insights into the best strategies for the community at large. Other institutions, individuals, and communities could also benefit from using this integrated footprint tool to make decisions. Using this tool to evaluate the impact of sustainability strategies allows stakeholders to see a broader range of indicators than would be apparent from examining the footprints separately. This additional knowledge allows stakeholders to evaluate the synergies and potential tradeoffs with each suggested strategy and make the best decisions depending on the institutions' goals.

## Supplementary Material

Supplemental data
