# Supplementary material for: Footprints in Action: How UVA Is Managing Its Sustainability Stewardship
Source: Sustain Clim Chang. 2023 Feb 23;16(1):48–63. doi: 10.1089/scc.2022.0067 (PMC9994435; doi:10.1089/scc.2022.0067)
Supplement: Supplemental data [file Supp_Data.docx]

**Supplemental Material: Footprints in Action: How UVA is Managing its Sustainability Stewardship**

**Part 1: Baseline data entered into IEFT**

The system bounds of the UVA GHG, N, P, and W footprints extend through all 550 of the academic, athletic, residential, and health system buildings. Data for each of the categories listed above (Table 2) are collected for all UVA-owned buildings within the City of Charlottesville and Albemarle County. Data are collected for the calendar year (January through December) for all scope 1 and 2 categories (stationary on-Grounds fuel sources, fleet, refrigerants, fertilizer, and electricity) as well as commuting (GHG and N), food (N), and wastewater (N) in scope 3. Food data are collected for all residential dining halls, catering, and small cafes operating on-Grounds that are university owned or commercial (ex: Subway operating on-Grounds). Food sold within concession stands is not included in the system bounds at this time due to inability to accurately capture all of the food served.

**Table S1:** Baseline data entered into the IEFT is listed under “category”, the amounts entered are listed under “usage data entered”, and units are listed under “units”. For the food, on-campus stationary, and commuting categories, the usage data are split into their respective sectors.

| Category | | Usage Data Entered (2016) | Units |
| --- | --- | --- | --- |
| Purchased Electricity | | 354,498,854.14 | Kilowatt hours (kwh) |
| Food | | | |
| Beans | | 187,614 | Kilograms (kg) |
| Beef | | 115,997 | Kilograms (kg) |
| Cheese | | 103,934 | Kilograms (kg) |
| Chicken | | 281,564 | Kilograms (kg) |
| Coffee and tea | | 22,210 | Kilograms (kg) |
| Eggs | | 172,499 | Kilograms (kg) |
| Fish | | 34,782 | Kilograms (kg) |
| Fruits | | 335669 | Kilograms (kg) |
| Grains | | 377,887 | Kilograms (kg) |
| Liquids | | 134,219 | Kilograms (kg) |
| Milk | | 323,192 | Kilograms (kg) |
| Nuts | | 14,938 | Kilograms (kg) |
| Oils | | 181,512 | Kilograms (kg) |
| Pork | | 98,459 | Kilograms (kg) |
| Potatoes | | 301,501 | Kilograms (kg) |
| Spices | | 10,324 | Kilograms (kg) |
| Sugars | | 99,877 | Kilograms (kg) |
| Vegetables | | 634,068 | Kilograms (kg) |
| On-campus stationary | | | |
| Coal (Steam Coal) | | 10,214.50 | Short Ton |
| Distillate Oil (#1-4) | | 100,496.18 | US gallon |
| LPG Propane | | 22,740.10 | US gallon |
| Natural Gas | | 1,193,129 | Million British Thermal Units (MMBtu) |
| Direct Transportation | | | |
| Gasoline Fleet | 176,290 | | US gallon |
| Diesel Fleet | 187,140 | | US gallon |
| B100 | 9,850 | | US gallon |
| Other liquid fuels | 32,413 | | US gallon |
| Commuting | | | |
| Car and Carpool | | 38,264,296.95 | Miles traveled |
| Commuter Rail | | 28,749.93 | Miles traveled |
| Public Bus | | 814,929.20 | Miles traveled |
| Agricultural Sources | | | |
| Fertilizer | | 4,462 | Pounds |
| Refrigerants and Chemicals | | | |
| HCFC-22 | | 109 | Pounds |
| HFC-134a | | 864 | Pounds |
| CFC-12 | | 43.74 | Pounds |
| R-410a | | 43.00 | Pounds |
| R-404a | | 301 | Pounds |
| Wastewater | | 287,063,700.00 | US gallons |
| Direct Water Use | | 515 | Millions of gallons |

**Part 2: Business as Usual (BAU) Calculations**

Slider bar changes for business as usual (BAU) growth between 2016 and 2025 are described here. BAU growth from 2016 to 2025 assumes standard growth and linear population.

Purchased Electricity was increased by 22% using estimated energy use projections.

Food was increased by 18% in each category (2% annual growth rate x 9 years = 18%).

Commuting and Direct Transportation Sources increased by 12%, corresponding to a projected 12% increase in population (faculty, staff, and students).

On-Campus Stationary Sources and Wastewater increased by 19%, corresponding to a projected 19% increase in gross square footage (total building space).

We did not adjust slider bars for Directly Financed Outsourced Travel, Agriculture Sources, Refrigerants & Chemicals, and Direct Water Use.

**Part 3: Food Reduction Scenarios**

**Explanation of food scenarios:**

1. **15% Vegetarian Meal Replacement:** Dining increases number of vegetarian meals served by 15% in all dining halls.
2. **Meat-focused café becomes plant-forward:** A meat-focused café or restaurant serves plant-forward meal options instead.
3. **Plant-forward (PF) Castle:** “The Castle” dining location serves plant-forward meal options.
4. **Plant-forward themed meals:** Three total plant-forward themed meals are served per month, one at each of the three dining halls at UVA applying to all the meal stations at the dining hall.
5. **Plant-forward Fridays in all dining halls:** One traditionally meat / animal-product focused station at each of the 3 main dining halls (Fresh Food Co., OHill, and Runk) will serve a plant-forward meal instead on every Friday.
6. **20% Beef replaced with chicken:** 20% of beef served in all dining locations will be replaced with chicken.
7. **Blended Burgers in all dining halls:** All dining hall burgers will be a blend of 80% beef and 20% mushrooms.
8. **Lean Path:** avoidable food waste measured from residential dining halls will be reduced by 50%.

**Health System Food Scenarios:**

1. **10% beef replaced with chicken:** 10% of all beef served within the UVA health system will be replaced with chicken.
2. **15% burgers replaced with Beyond Burgers:** 15% of beef patty burgers served in the UVA health system will be replaced with plant-based “Beyond Burgers.”
3. **Mindful Mondays:** The UVA health system will serve meatless options on Mondays.
4. **One station has no beef for 6 months:** One of the eight stations in the UVA health system cafeteria that traditionally serves beef will stop serving beef for 6 months.

**Table S2a**. Table showing slider bar changes for each food category and each food reduction scenario (see descriptions above). The percentages listed in the table illustrate the percentage change in each category from BAU 2025, converted to a 2016 percentage scale.

| **Food Category** | **A. 15% Vegetarian Meal Replacement** | **B. Meat-focused café becomes PF** | **C. PF Castle** | **D. PF themed meals** | **E. PF Fridays** | **F. 20% Beef replacement** | **G. Blended Burgers** | **H. Lean Path** |
| --- | --- | --- | --- | --- | --- | --- | --- | --- |
| Spices |  |  |  |  |  |  |  |  |
| Sugars |  |  |  |  |  |  |  |  |
| Oils |  |  |  |  |  |  |  |  |
| Coffee and tea |  |  |  |  |  |  |  |  |
| Liquids |  |  |  |  |  |  |  |  |
| Nuts | +3.8% |  |  |  |  |  |  |  |
| Beans | +3.8% | +1.8% | +2.1% |  |  |  |  |  |
| Potatoes | +3.8% |  |  |  |  |  |  |  |
| Fruits | +3.8% |  |  |  |  |  |  | -1.6% |
| Vegetables | +3.8% | +0.52% | +0.63% |  |  |  | +0.42% | -1.6% |
| Grains | +3.8% |  |  |  |  |  |  | -0.8% |
| Eggs | +3.8% |  |  |  |  |  |  |  |
| Cheese | +3.8% |  |  |  |  |  |  |  |
| Milk | +3.8% |  |  |  |  |  |  |  |
| Fish | -17.7% | +3.2% | +3.8% | -1.3% | -0.46% |  |  |  |
| Chicken | -17.7% | +0.78% | -1% | -1.6% | -0.58% | +9.7% |  | -0.34% |
| Pork | -17.7% |  | -5% | -1.9% | -0.67% |  |  |  |
| Beef | -17.7% | -8.5% | -1.3% | -1.2% | -0.45% | -23.6% | -2.2% | -0.55% |

**Table S2b**. Table showing slider bar changes for each food category and each food reduction scenario from the UVA Health System. The percentages listed in the table illustrate the percentage change in each category from BAU 2025, converted to a 2016 percentage scale. The last column includes the 18% growth from 2016 to 2025, and reflects the percentage (rounded to the nearest whole percent) that was entered into the IEFT.

| **Food Category** | **I. 10% Beef replacement** | **J. 15% replaced with Beyond Burgers** | **K. Mindful Monday** | **L. One station with no beef** | **Total (all Food Scenarios)** | ***Rounded Total (incl. 18% growth to 2025)** |
| --- | --- | --- | --- | --- | --- | --- |
| Spices |  |  |  |  |  | +18% |
| Sugars |  |  |  |  |  | +18% |
| Oils |  |  |  |  |  | +18% |
| Coffee and tea |  |  |  |  |  | +18% |
| Liquids |  |  |  |  |  | +18% |
| Nuts |  |  |  |  | +3.8% | +22% |
| Beans |  |  | +1.3% |  | +9% | +27% |
| Potatoes |  |  |  |  | +3.8% | +22% |
| Fruits |  |  |  |  | +2.3% | +20% |
| Vegetables |  | +0.35% | +1.5% |  | +5.7% | +24% |
| Grains |  |  | +1.7% |  | +4.7% | +23% |
| Eggs |  |  |  |  | +3.8% | +22% |
| Cheese |  |  |  |  | +3.8% | +22% |
| Milk |  |  | +1.75% |  | +5.6% | +24% |
| Fish |  |  | -5% |  | -17.5% | 0% |
| Chicken | +1.5% |  | -4.5% |  | -13.8% | +4% |
| Pork |  |  | -3.9% |  | -29.1% | -11% |
| Beef | -3.7% | -1.9% | -5.1% | -2.3% | -68.6% | -51% |

**Table S3**. This table shows the output from the “Food” tab of the IEFT tool when all food reduction scenarios are implemented (when the rounded total percentages from Table S2b are entered into the tool).

| **Category** | **C Footprint (kg C equivalents)** | **N Footprint (kg)** | **P Footprint (kg)** | **Blue Water Footprint (m^3^)** | **Green Water Footprint (m^3^)** | **Blue/Green Water Footprint (m^3^)** |
| --- | --- | --- | --- | --- | --- | --- |
| Beans | 185850 | 1581 | 722 | 75245 | 298103 | 373348 |
| Beef | 1503376 | 18975 | 6369 | 29840 | 735091 | 764931 |
| Cheese | 1240101 | 12403 | 1544 | 23458 | 255374 | 278832 |
| Chicken | 1478772 | 35434 | 8170 | 54758 | 506003 | 560762 |
| Coffee and tea | 9435 | 1620 | 16 | 4908 | 397026 | 401934 |
| Eggs | 744987 | 13468 | 4573 | 27358 | 253801 | 281159 |
| Fish | 133215 | 2886 | 0 | 0 | 0 | 0 |
| Fruits | 145009 | 1191 | 245 | 62927 | 74457 | 137384 |
| Grains | 399729 | 8336 | 2409 | 44453 | 367821 | 412274 |
| Liquids | 163130 | 860 | 96 | 4751 | 41179 | 45930 |
| Milk | 537016 | 8241 | 1020 | 24045 | 259290 | 283336 |
| Nuts | 21323 | 249 | 55 | 38076 | 24118 | 62194 |
| Oils | 349120 | 58 | 649 | 21407 | 341821 | 363228 |
| Pork | 602010 | 12007 | 2982 | 56521 | 359453 | 415974 |
| Potatoes | 77244 | 2312 | 666 | 32393 | 19758 | 52151 |
| Spices | 8893 | 340 | 27 | 365 | 914 | 1279 |
| Sugars | 109605 | 227 | 72 | 3675 | 14685 | 18360 |
| Vegetables | 573959 | 7070 | 1717 | 76294 | 70559 | 146853 |

**Food Reduction Scenario Calculations**

All numbers presented in written calculations are reported using four significant figures.

**Conversion from 2025 to 2016 scale**

All food slider bar units were based on 2016 data entered into the IEFT tool (Leach et al., 2020). If we wanted to show a 15% reduction from the projected 2025 number, the percentage would have to be converted to the 2016 percentage scale in order to represent the same absolute number (see example below).

Example:

2016 Baseline for beef: 200 kg

2025 Baseline for beef: 300 kg

We want to reduce beef by 15% in 2025.

A 15% reduction in 2025 means a reduction of ($\text{0.15 × 300}$) = 45 kg.

To represent the 45 kg reduction on the 2016 scale, we need to convert it based on the 2016 baseline number.

45 kg divided by the 2016 number = $\frac{\text{45 kg}}{\text{200 kg}}$ = 22.5% reduction on the 2016 scale.

For food scenarios, all 2025 reduction numbers were converted to a 2016 percentage scale in this way.

**15% Vegetarian Meal Replacement (Scenario A)**

For this scenario, all meats (fish, chicken, pork, beef) were reduced by 15% from 2025 numbers, while nuts, beans, potatoes, fruits, vegetables, grains, eggs, cheese, and milk were increased by 3.25% from 2025 numbers to ensure the same weight of food was replaced with vegetarian products.

After converting to 2016 percentages, meats were reduced by 17.7% while all other aforementioned categories were increased by 3.83% from 2025 numbers.

**Meat-Focused Café becomes Plant-Forward (Scenario B)**

For this scenario, beef data were taken from a meat-focused café at UVA (8390 kg of beef). To turn it into a plant-based meal, 1/3 of the beef weight was replaced with vegetables, 1/3 was replaced with beans, 1/9 was replaced with fish, and 2/9 was replaced with chicken. The appropriate reductions and increases were added to the respective food categories, and the overall percent changes in each category were calculated.

The original weight of food served at the location was 28,170 kg, with beef making up about 30% of the overall amount of food. After the replacement scenario, the amount of food remained the same, but all of the beef was replaced as listed above. After the scenario was implemented, fish made up 3% of the total food weight, chicken made up 6% of the total food weight, and the rest was made up of vegetarian products. This resulted in new totals of 107,600 kg of beef, 283,400 kg of chicken, 35,710 kg of fish, 638,000 kg of vegetables, and 190,400 kg of beans. The percent change in each food category was calculated and converted to 2016 numbers.

**Plant-Forward Castle (Scenario C)**

For this scenario, beef (1234 kg), chicken (4798 kg), and pork (4175 kg) data were taken from The Castle, a dining location at UVA. To turn it into a plant-based meal, 1/3 of the amount of beef was replaced with vegetables, 1/3 was replaced with beans, 1/9 was replaced with fish, and 2/9 was replaced with chicken. These same substitutions were also conducted for the original pork amounts and chicken, leaving 2/9^th^ of the original chicken weight remaining. The appropriate reductions and increases were added to the respective categories, and the overall percent changes in each category were calculated. Similar to the Plant-Forward scenario, the total weight of food (10,840 kg) reminded the same. This resulted in new totals of 114,800 kg of beef, 279,000 kg of chicken, 35,910 kg of fish, 94,270 kg of pork, 637,500 kg of vegetables, and 191,000 kg of beans. The percent change in each food category was calculated and converted to 2016 numbers.

**Plant-Forward Fridays and Plant-Forward Themed Meals (Scenarios D and E)**

For these scenarios, “Plant-Forward” meals were considered to have 2 ounces of meat, and each dining hall was considered to have an average of 7 food stations.

The number of meal swipes used across all dining halls for one week were obtained based on 2016 data (1,735,000), as well as the average weight of meat per meal swipe (4.5 oz). The percentage reduction needed to reach 2 oz meat per meal swipe was calculated (-56%).

The total amounts of beef (39,020 kg), chicken (121,500 kg), fish (12,060 kg), and pork (49,490 kg) served across all dining halls for one week were obtained.

**For PF Fridays,** the total amounts of beef, chicken, fish, and pork were divided by (7 days per week × 7 food stations) to find the amounts of beef (796 kg), chicken (2479 kg), fish (246.2 kg), and pork (1010 kg) that would be served on one day at one food station at every dining hall. These amounts were then multiplied by 56% to find the reduction amount needed in each category to reach 2 oz of meat per meal. The reduction amount for each category was then divided by the 2016 baseline amount to find the reduction percentage. This reduction percentage was then converted from the 2025 to the 2016 scale.

**For PF Themed Meals,** the total amounts of beef, chicken, fish, and pork were divided by (7 days per week × 3 dining halls × 3 meals per day) and then multiplied by (3 days that PF Themed Meals would be implemented). This calculated the amounts of beef (1858 kg), chicken (5784 kg), fish (574.4 kg), and pork (2357 kg) that were served at all stations of one dining hall for 3 meals per month. These amounts were then multiplied by 56% to find the reduction amount needed in each category to reach 2 oz of meat per meal. The reduction amount for each category was then divided by the 2016 baseline amount to find the reduction percentage. This reduction percentage was then converted from the 2025 to the 2016 scale.

**All Beef Replacement with Chicken by weight Scenarios (Scenarios F and I)**

**For 20% beef replaced with chicken,** the baseline 2016 amounts for beef (116,000 kg) and chicken (281,600 kg) were obtained. The amount of beef in a 20% reduction was calculated, and an equivalent weight of chicken was added to the 2016 chicken amount. This resulted in final amounts of 92,800 kg of beef and 304,800 kg of chicken. The percent increase in chicken was calculated. The percent increase in chicken as well as the 20% decrease in beef were then applied to 2025 numbers and converted to 2016 percentages.

**For the Health System 10% beef replaced with chicken,** the baseline 2016 amounts for beef (36,020 kg) and chicken (77230 kg) within the health system were obtained. The amount of beef in a 10% reduction was calculated (3602 kg), and an equivalent amount of chicken was then added to the overall UVA 2016 chicken amount. This resulted in an overall total of 285,200 kg chicken. The percent increase in the overall 2016 chicken amount was calculated. The amount of beef in a 10% health system reduction was subtracted from the overall 2016 beef amount, leading to an overall total of 112400 kg of beef. The percent decrease in the overall 2016 beef amount was calculated. The percent increase in chicken as well as the percent decrease in beef were then applied to the 2025 numbers and converted to 2016 percentages.

**Blended Burgers in all Dining Halls (Scenario G)**

The term “blended burgers” refers to burgers made up of 80% ground beef and 20% mushrooms in place of 100% beef burgers. These blended burgers replaced all burgers served in 4 dining halls at UVA (10,910 kg). 20% of the total weight of burgers served was subtracted from the total beef at UVA (2,182 kg). This was then replaced with the same weight (kg) of vegetables added back into the total weight of vegetables served in 2025. This led to final amounts of 113,800 kg of beef and 636,300 kg of vegetables. The percent changes were then calculated and converted to 2016 percentages.

**Lean Path (Scenario H)**

Food waste produced in UVA dining halls was measured beginning in January 2017. The annual amounts of back of house food waste in residential dining halls were measured for the calendar year (1/1/2017 to 12/31/2017) using the LeanPath system. The system classified food waste into 10 categories (trim waste, overproduction, post-consumer, expired, overcooked, spoiled, handling, quality, catering guarantee, and equipment failure). All categories except trim waste (which was the largest contributor to the total weight of waste) were considered avoidable, totaling 33,580 kg of food. In this scenario, we reduced the amount of avoidable waste by 50% (16,790 kg). We then distributed this reduction in food weight proportionally across the following food categories: vegetables (reduced by 8,395 kg), fruit (reduced by 4,468 kg), grains (reduced by 2,573 kg), poultry (reduced by 812 kg), and beef (reduced by 541.5 kg). These reductions were applied to the overall food categories and the percent decrease was calculated and converted to 2016 percentages.

**15% Burgers Replaced with Beyond Burgers in Health System (Scenario J)**

The total amount of beef consumed in the health system during 2016 was calculated (12,740 kg). Since Beyond Burgers are primarily made up of vegetables, it was assumed that all Beyond Burger replacements would lead to an increase in the vegetable category only. 15% of the total amount of beef was calculated and replaced with an equivalent amount of vegetables (1,911 kg). This amount was subtracted from the baseline amount of beef and added to the baseline amount of vegetables, leading to final amounts of 114,100 kg of beef and 636,000 kg of vegetables. The percent change for each category was calculated and converted to 2016 percentages.

**Mindful Mondays in the Health System (Scenario K)**

For this scenario, Mindful Mondays are one day during the week when the health system serves only vegetarian options. It was assumed that a normal meal contained meat in it. The total number of “meat meals” in one year was calculated as 365 days x 3 meals per day = 1095 meals. The total number of Mondays per year is 52, and the total number of meals consumed on Mondays is 52 days x 3 meals per day = 156 meals. To find the percent of meat meals replaced with vegetarian meals for Mindful Mondays, we calculated 156 meals / 1095 meals = 14%. All health system totals for beef (36020 kg), chicken (77230 kg), fish (10710 kg), and pork (23090 kg) were obtained, and each category was reduced by 14%. These reductions were then applied to the overall UVA totals for each category. The total weight of meat that was reduced (20,590 kg) was then redistributed proportionally according to an example vegetarian meal consisting of 0.12 kg vegetables, 0.08 kg grains, 0.03 kg beans, and 0.07 kg milk products. The proportional amounts were added to the overall UVA totals for these four categories. The percent change for each category was calculated and converted to 2016 percentages.

**One Station has no Beef for 6 Months in the Health System (Scenario L)**

The total weight of beef served at 1/8 of the stations in the health system was eliminated for 6 months out of the year, resulting in a reduction of 2,251 kg. The beef weight was subtracted from the UVA total, resulting in a new total of 113,700 kg of beef. The percent decrease from the baseline was calculated and converted to 2016 percentages.

**Table S4**. Table showing exact baseline 2016 and BAU 2025 data that were used for the above food calculations.

| **Food Category** | **2016 (kg)** | **BAU 2025 (kg)** |
| --- | --- | --- |
| Beans | 187,613 | 221,384 |
| Beef | 115,996 | 136,876 |
| Cheese | 103,934 | 122,642 |
| Chicken | 281,563 | 332,245 |
| Coffee and tea | 22,209 | 26,207 |
| Eggs | 172,498 | 203,548 |
| Fish | 34,781 | 41,042 |
| Fruits | 301,500 | 355,770 |
| Fruits | 335,668 | 396,088 |
| Grains | 377,886 | 445,906 |
| Liquids | 134,219 | 158,378 |
| Milk | 323,192 | 381,366 |
| Nuts | 149,38 | 17,627 |
| Oils | 181,511 | 214,183 |
| Pork | 984,59 | 116,182 |
| Potatoes | 301,500 | 355,770 |
| Spices | 10,323 | 12,182 |
| Sugars | 99,877 | 117,855 |
| Vegetables | 634,068 | 748,200 |

**Part 4: Energy Reduction Scenarios**

**Explanation of Energy Scenarios:**

1. **Fuel optimization:** Coal usage at the heat plant would be removed and switched to natural gas use.
2. **Heat plant efficiency improvements:** Thermal efficiency of the Main Heating Plant was increased from 80-82% to 83-85%.
3. **Distribution efficiency / Low Temperature Hot Water (LTHW):** UVA operates an existing district heating system centered on the Main Heating Plant, which produces saturated steam and medium temperature hot water (MTHW) for campus heating and other uses. LTHW generation and distribution systems can be more efficient than steam systems and even MTHW, because energy generation is much more efficient at lower temperatures and less heat is lost in distribution between the points of generation and consumption. Furthermore, LTHW systems can take advantage of more efficient technologies such as waste heat recapture that then reduces electrical and water consumption.
4. **Chilled water plant efficiency improvements:** Electricity and water are used to generate 42^o^F water, which is used to provide air conditioning to UVA facilities. Chiller plants consume more than 20% of the purchased electricity and 25% of the water at UVA, so it is a primary target for energy and water efficiency and N reduction.
5. **Dominion Electricity Grid improvements:** Emission factors for purchased electricity take into account the fossil fuels used to generate the electricity. While emission factors for purchased electricity in this area of the country have remained relatively stable since 2010, it is anticipated that Dominion will decrease their use of coal in electricity generation and increase their renewable energy or use of natural gas between 2017 and 2025.
6. **On-grounds solar:** UVA is working with consultants to develop and identify optimal locations and funding mechanisms for additional building-mounted PV and solar thermal projects on Grounds.
7. **Off-grounds utility scale solar (UVA Hollyfield):** UVA and its Darden School of Business have entered into a solar power partnership with Dominion Virginia Power. The UVA Hollyfield Solar project, owned by Dominion, who will construct and operate it, is expected to produce an estimated 17 megawatts of alternating current (AC) power, a figure representing about 10% of the University’s electrical demand.
8. **Off-grounds utility scale solar (UVA Puller):** Similar to other off-grounds utility scale projects listed above.
9. **Additional off-grounds utility scale solar:** UVA continues to explore other utility-scale solar opportunities and seeks to add another utility-scale renewable project in the future. UVA will also actively pursue additional installations and will move forward as funding allows.
10. **Existing building improvements:** To successfully achieve energy reductions across the building portfolio, projects will be identified and implemented as systematic technology rollouts, as building-specific optimization, or through enhanced preventative maintenance activities.
11. **Green IT:** Computer use and IT infrastructure are extensive throughout UVA, consuming an estimated 5-15% of all electricity on-grounds, but the diffuse operation and management of IT equipment creates a challenge for assessing and understanding the N footprint. Through the Green IT Working Group, other inter-departmental IT committees and task forces, and the Office for Sustainability, UVA will begin cataloging computing systems across Grounds as a first step toward assessing opportunities for improving efficiency
12. **Outreach and Engagement:** Outreach and engagement programs aimed at reducing energy use on-Grounds have been in existence for over a decade. The 2016-2020 UVA Sustainability Plan includes a goal to further increase sustainability awareness and outlines educational strategies to expand programs that translate awareness into action.
13. **Gasoline fleet improvements:** UVA has started assessing the fleet through a LEAN Initiative for Facilities Management. Through this process, UVA has laid groundwork that will result in improved processes, reduced waste, and better use of resources. The end goal is to define a high-value fleet that allows UVA staff to perform their duties efficiently, with the least amount of fuel consumed.
14. **Diesel fleet improvements:** Parking and Transportation has developed a long-range fleet replacement plan that will result in more fuel-efficient buses that are built to adhere to more recent emission regulations issued by the Environmental Protection Agency. As new buses are put into service, older and less fuel-efficient buses will be retired. University Transit System (UTS) buses are also equipped with various levels of technology that allow Parking and Transportation to evaluate idle times and passenger count trends. These technologies could be used to improve route efficiency, reduce idling, and optimize vehicle miles traveled.
15. **Green Building Standards:** UVA continues to expand research activities, medical services, patient visits, academic offerings, and associated support services. To meet this growth, there are extensive and continuous construction projects to build and renovate new and existing facilities. The additional space presents opportunities to improve energy efficiency and sustainability while simultaneously adding to the GHG footprint of the University. UVA developed Green Building Standards (GBS) under the Facility Design Guidelines that prescribe minimum requirements for all new construction and major renovation projects. The GBS embed requirements for equipment efficiency as well as design guidelines based on best practices for energy efficiency at UVA. The most significant element that will affect the energy efficiency of new and renovated buildings on-Grounds is a requirement to achieve a minimum 25 percent reduction in energy use intensity (EUI) as compared to an EUI of a similar usage type building, to be determined by UVA. Projects will also be required to evaluate the feasibility of deeper energy reductions and demonstrate that the project analyzed options through energy modeling and life cycle costing.
16. **Green Building Standards (Stretch):** These are additional reductions for the goal for the Green Building Standards.
17. **Green Labs:** UVA’s research activities and medical testing laboratories require energy-intensive infrastructure. There are a multitude of facilities on-Grounds, which house research and medical testing with varying requirements for handling hazardous chemicals and biological agents. Additionally, the laboratories require power-hungry equipment to preserve and support research. The energy reduction approach with UVA’s laboratory spaces is right-sizing equipment and infrastructure relative to the need of the Principal Investigator’s research.

**Table S5**. Table showing slider bar changes for each applicable energy category and each energy reduction scenario (see descriptions above). The percentages listed in the table illustrate the percentage change in each category from BAU 2025. The last column includes growth from 2016 to 2025, and reflects the percentage (rounded to the nearest whole percent) that was entered into the IEFT. Purchased Electricity and Direct Transportation Sources were entered into the “C, N, P, W Footprints” tab, while the On-Campus Stationary Sources were entered into the “On-Campus Stationary Sources” tab.

| **Energy Category** | **A** | **B** | **C** | **D** | **E** | **F** | **G** | **H** | **I** |
| --- | --- | --- | --- | --- | --- | --- | --- | --- | --- |
| Purchased Electricity |  |  |  | -3% | -4% | -1% | -10% | -9% | -25% |
| Direct Transportation Sources |  |  |  |  |  |  |  |  |  |
| On Campus Stationary Sources (Coal) | -100% |  |  |  |  |  |  |  |  |
| On Campus Stationary Sources (Natural Gas)* | +22% | -3% | -1% |  |  |  |  |  |  |

| **Energy Category** | **J** | **K** | **L** | **M** | **N** | **O** | **P** | **Q** | **Total** | ***Rounded Total (incl. growth to 2025)** |
| --- | --- | --- | --- | --- | --- | --- | --- | --- | --- | --- |
| Purchased Electricity | -15% | -1% | -2% |  |  | -3% | -3% | -2% | -78% | -56% |
| Direct Transportation Sources |  |  |  | -8.5% | -7.9% |  |  |  | -16.4% | -4% |
| On Campus Stationary Sources (Coal) |  |  |  |  |  |  |  |  | -100% | -100% |
| On Campus Stationary Sources (Natural Gas) |  |  |  |  |  |  |  |  |  | +39% |

*For natural gas, scenario A was implemented in 2016, then 19% growth to BAU 2025 was applied, then scenarios B and C were applied.

**Table S6**. This table shows the output from the “C, N, P, W Footprints” tab of the IEFT tool when all energy reduction scenarios are implemented (when the rounded total percentages from Table S5 are entered into the tool).

| **Category** | **C Footprint (Metric Tons C equivalents)** | **N Footprint (Metric Tons)** | **P Footprint (Metric Tons)** | **W Footprint (m^3^)** |
| --- | --- | --- | --- | --- |
| Purchased Electricity | 61052 | 13 | 0 | 2815 |
| Food | 10665 | 161 | 42 | 631264 |
| On-Campus Stationary Sources | 93322 | 24 | 0 | 0 |
| Direct Transportation Sources | 3716 | 3 | 0 | 96961 |
| Commuting | 44812 | 27 | 0 | 0 |
| Directly Financed Outsourced Travel | 0 | 0 | 0 | 0 |
| Agriculture Sources | 17 | 1 | 0 | 0 |
| Refrigerants & Chemicals | 2501 | 0 | 0 | 0 |
| Wastewater | 159 | 2 | 1 | 0 |
| Direct Water Use | 0 | 0 | 0 | 1948729 |

**Energy Reduction Scenario Calculations**

All numbers presented in written calculations are reported using four significant figures.

**On Campus-Stationary (Scenarios A, B, C)**

Scenario A is the fuel optimization scenario, where coal usage is reduced by 100%. Scenario A is meant to be implemented first, immediately, while Scenarios B and C are long-term efficiency improvements. The baseline amount of coal (10,210 short tons) was converted to MMBtu using the conversion factor 26.28 MMBtu/short ton, giving 268,400 MMBtu. The baseline amount of coal was then reduced by 100%. To calculate a replacement of coal with natural gas, the amount of coal reduced (i.e. all of it) was added to the baseline amount of natural gas, giving 1,462,000 MMBtu of natural gas. The reduced coal amount and the increased natural gas amounts were both projected to grow 19% between 2016 and 2025, giving 0 MMBtu coal in 2025 and 1,735,000 MMBtu natural gas in 2025. We then implemented Scenarios B and C, which involved a 3% reduction from the new natural gas value, and a 7% reduction from the new value for coal. Since Scenario A involved a complete elimination of coal, the 7% reduction in coal was converted to natural gas to calculate its effects. 7% of the 2016 coal baseline would have been 18,790 MMBtu. If we reduced natural gas by an additional 18,790 MMBtu, that would lead to an additional 1.08% reduction in natural gas. Thus, we reduced natural gas by 3% + 1.08% = 4.08% in total. After all three scenarios were implemented, they resulted in 0 MMBtu coal and 1,664,000 MMBtu natural gas. The final percent changes to enter into the IEFT were then calculated based on change from 2016, resulting in a 100% decrease in coal and a 39% increase in natural gas.

**Direct Transportation Sources (Scenarios M, N)**

The reduction scenarios that affected direct transportation sources were scenarios M and N – gasoline and diesel fleet improvements, respectively. For both of these scenarios, we obtained the baseline 2016 activity in US gallons. The gasoline fleet baseline amount was 176,300 US gallons. The diesel fleet baseline amount was 187,100 US gallons. We then converted these values to MMBtu using the conversion factors 0.1209 MMBtu/US gallon of gasoline and 0.1376 MMBtu/US gallon of diesel. The 2016 baseline values in MMBtu were 21,310 for the gasoline fleet, and 25,750 for the diesel fleet. These values were then projected to 2025 activity (increased by 12%), giving 23,780 MMBtu for the gasoline fleet and 28,720 MMBtu for the diesel fleet. The scenario called for a 25% reduction in both categories, so we calculated a 25% reduction from 2025 for each category. This led to final amounts of 17,830 MMBtu for the gasoline fleet and 21,540 MMBtu for the diesel fleet. We added these reduced gasoline and diesel fleet values to the rest of the Direct Transportation Sources (B100 Fleet and Other Liquid Fuels), giving a total of 76,940 MMBtu for Direct Transportation Sources (see Table S7). We then calculated the overall percent reduction in Direct Transportation Sources based on the 2016 scale, resulting in a 16% decrease in Direct Transportation Sources.

**Purchased Electricity (all other scenarios)**

All percentages for Purchased Electricity were obtained from the SIMAP template and tool and entered directly into the tool, including the 22% growth from 2016 to 2025.

**Table S7.** Table showing exact baseline 2016 and BAU 2025 data in MMBtu that were used for the above energy calculations.

| **Food Category** | **2016 (MMBtu)** | **BAU 2025 (MMBtu)** |
| --- | --- | --- |
| Purchased Electricity | 1,957,568 | 2,388,233.76 |
| Direct Transportation Sources (B100 Fleet) | 1,260 | 1,406 |
| Direct Transportation Sources (Diesel Fleet) | 25,745 | 28,721 |
| Direct Transportation Sources (Gasoline Fleet) | 21,314 | 23,778. |
| Direct Transportation Sources (Other [Liquid Fuels]) | 32,413 | 36,159 |
| On-Campus Stationary Sources (Coal) | 268,396 | 318,532 |
| On-Campus Stationary Sources (Natural Gas) | 1,193,128 | 1,416,005 |

**Part 5: Wastewater Reduction Scenario**

The only wastewater reduction scenario is a stormwater retention scenario. This stormwater retention scenario includes implementing strategies to reduce the N content of stormwater before reaching the Rivanna Wastewater Treatment Plant, which will in turn reduce the amount of N the university is producing via wastewater.

The scenario was included in the N Action Plan but it has only a 0.07% reduction, minimally affecting all footprints. As a result, it was not included in the IEFT calculations.

**Part 6: Summary of Projections and Scenarios**

**Table S8:** The category impacted, projected business-as-usual (BAU) percent increase from 2016 to 2025, and percent reduction in activity due to the action plan scenarios. The percentages were calculated in Microsoft Excel, based on scenarios listed in Table 2. The resulting percentages listed in the third column were entered into the IEFT for each category to examine impacts on UVA’s environmental footprints. More information on how these percentages were calculated can be found in the supplementary material (Table S1-S7).

| **Categories** | **Projected BAU increase from 2016 to 2025** | **Projected impact of action plan scenarios on 2025 BAU** |
| --- | --- | --- |
| Purchased Electricity | 22% | -56% |
| Direct Transportation Sources | 12% | -4% |
| All commuting slider bars | 12% | 12% |
| Wastewater | 19% | 19% |
| Direct water use | 0% | 0% |
| Refrigerants and Chemicals | 0% | 0% |
| Agricultural Sources | 0% | 0% |
| *On-campus stationary* | | |
| Coal | 19% | -100% |
| Distillate Oil (#1-4) | 19% | 19% |
| LPG (Propane) | 19% | 19% |
| Natural Gas | 19% | 39% |
| *Food* | | |
| Spices | 18% | 18% |
| Sugars | 18% | 18% |
| Oils | 18% | 18% |
| Coffee and tea | 18% | 18% |
| Liquids | 18% | 18% |
| Nuts | 18% | 22% |
| Beans | 18% | 27% |
| Potatoes | 18% | 22% |
| Fruits | 18% | 20% |
| Vegetables | 18% | 24% |
| Grains | 18% | 23% |
| Eggs | 18% | 22% |
| Cheese | 18% | 22% |
| Milk | 18% | 24% |
| Fish | 18% | 0% |
| Chicken | 18% | 4% |
| Pork | 18% | -11% |
| Beef | 18% | -51% |

**Part 7: Nitrogen Damage Costs**

Nitrogen damage costs can be further split by type (N to water, NH_3_, N_2_O, and NO_x_) (figure S1) and by media (coastal water, groundwater, freshwater, terrestrial, and atmospheric) (figure S2) using estimates from Birch et al. 2014. The same scenarios were used in the main text Table 4.


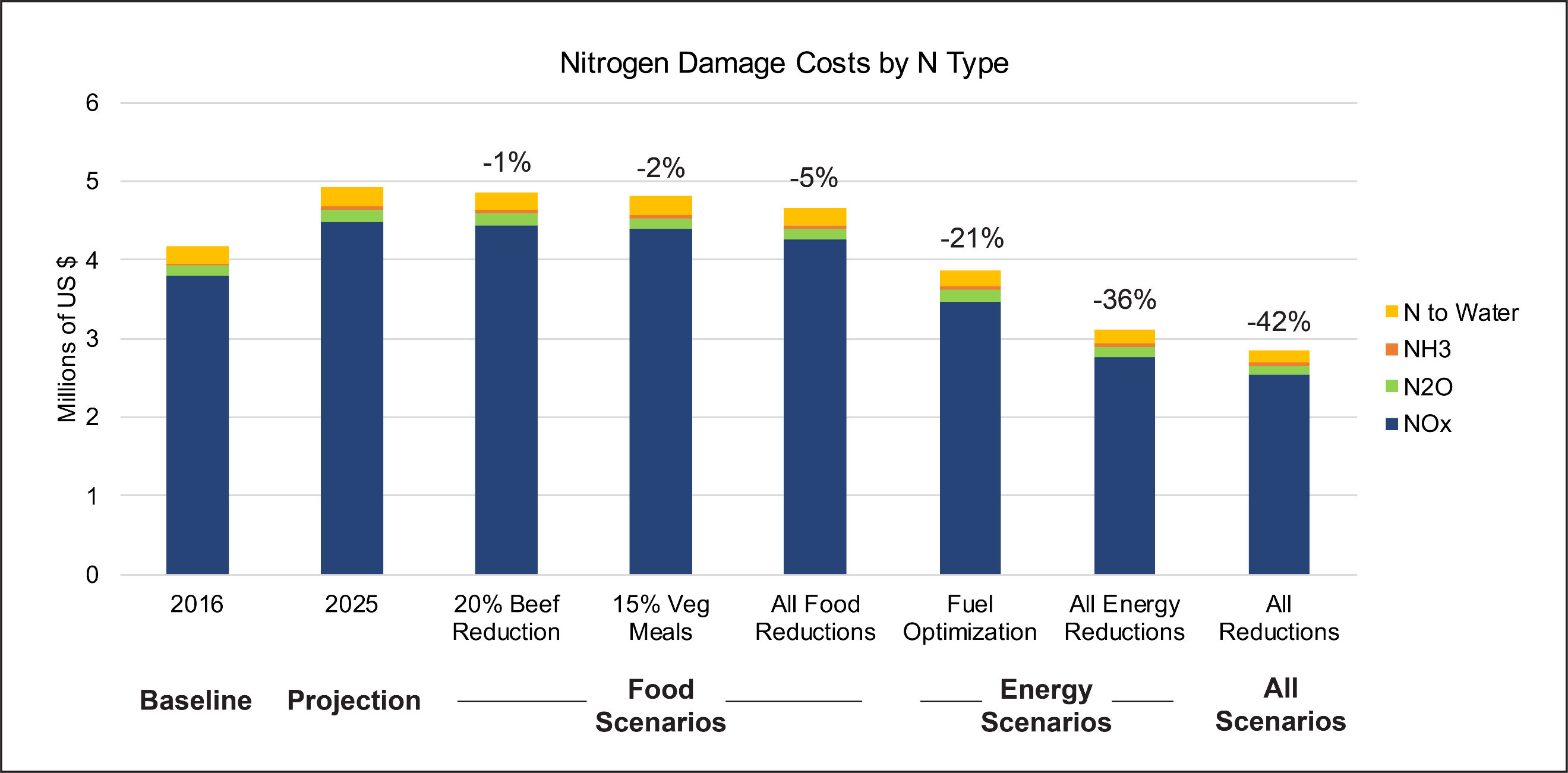


**Figure S1:** Depicts the estimated damage costs by sector for N by N type. The estimated damage cost reductions are shown above each bar for the 2016 baseline, 2025 projection year, individual food scenarios, individual energy scenarios and all sets of scenarios combined. These damage costs are aggregated by category impacted (N to water, NH_3_, N_2_O, and NO_x_).


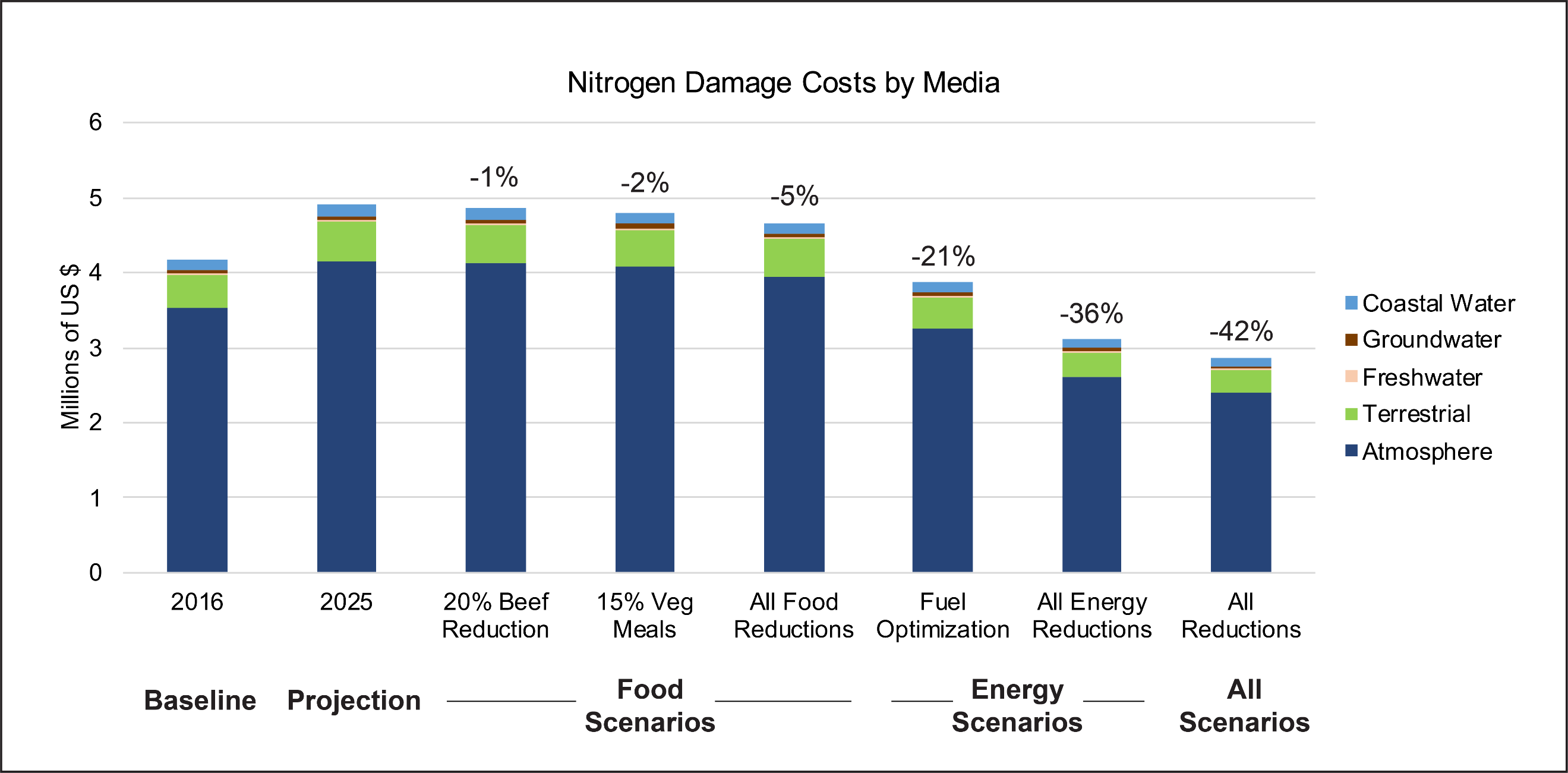


**Figure S2:** Depicts the estimated damage costs by sector for N by media. The estimated damage cost reductions are shown above each bar for the 2016 baseline, 2025 projection year, individual food scenarios, individual energy scenarios and all sets of scenarios combined. These damage costs are aggregated by category impacted (coastal water, groundwater, freshwater, terrestrial, and atmosphere).

**Table S9**

|  |  |  |  |
| --- | --- | --- | --- |
| **Media where impact is observed** | **Damage or benefit associated with reactive N emission** | **US damage costs ($/kg N)** | **Data source** |
| **Atmosphere** | Human respiratory health – NOx (Health/Social) | $ 32.20 | Birch et al. 2011 |
|  | Human respiratory health - NH_3_ (Health/Social) | $ 6.87 | Birch et al. 2011 |
|  | Visibility - NOx, NH_3_ (Ecosystems) | $ 0.43 | Birch et al. 2011 |
|  | Greenhouse gas damages - N_2_O (Climate) | $ 15.07 | van Grinsven et al. 2013 |
|  | Climate cooling benefits - NOx, NH3 (Climate) | $ (5.02) | van Grinsven et al. 2013 |
| **Terrestrial** | Damage to structures - NOx, NH3 (Health/Social) | $ 0.12 | Birch et al. 2011 |
|  | Ozone damage to crops – NOx (Agriculture) | $ 2.11 | Birch et al. 2011 |
|  | UV damage to crops - N_2_O (Agriculture) | $ 1.48 | van Grinsven et al. 2013, Compton et al. 2011 |
|  | Ozone damage to forests – NOx (Ecosystems) | $ 1.24 | Birch et al. 2011 |
| **Aquatic - Freshwater** | Lake waterfront property values (Ecosystems) | $ 0.23 | Dodds et al. 2009 |
|  | Recreational Freshwater use (Ecosystems) | $ 0.19 | Dodds et al. 2009 |
|  | Endangered species protection (Ecosystems) | $ 0.01 | Dodds et al. 2009 |
| **Groundwater** | Replacement with bottled water (Health/Social) | $ 0.15 | Kusiima & Powers 2010 |
|  | Treatment of private well water (Health/Social) | $ 0.60 | Compton et al. 2011 |
| **Aquatic - Coastal** | Recreational use of estuary (Ecosystems) | $ 7.11 | Birch et al. 2011 |

Damages associated with nitrogen inputs to the following environmental classes. Damage costs are presented as the USD of damage associated with one kilogram of N. Most of the US numbers are derived from national or regional assessments. Negative numbers represent benefits.

**Part 8: Equations for GHG, N, P, and W footprints**

**Definitions**

GWP file has all of the global warming potentials for refrigerants and chemicals

MT = Metric Tons

MTCDE = Metric Tons CO_2_ Equivalents

C = Carbon

N = Nitrogen

P = Phosphorus

W = Water

SW = Surface Water

GW = Ground Water

**Food Calculations**

Food Carbon Footprint (kg) = Food Inventory Value × C Food Footprint (in table provided)

Food Nitrogen Footprint (kg) = N Food Waste + N Virtual

N Food Waste (kg) = Food Waste (%) × N Food Supply (kg)

N Virtual = N Food Supply (kg) * Conventional Virtual N Factor (kg N loss / kg N food)

N Food Supply (kg) = Inventory (kg) * N Content (Unitless)

Food P Footprint (kg) = Inventory Value (kg) * P Food Footprint (in table provided)

Food Blue Water Footprint (m3) = Blue Water Footprint (m^3^)

Food Green Water Footprint (m3) = Blue Green Footprint (m^3^)

Food Blue Green Water Footprint (m3) = Blue Green Water Footprint (m^3^)

**C and N Calculations**

CO2_r = Inventory * CO2 Emission Factor

CO2 (MTCDE) = CO2_r / 1000

eCO2_r = inventory * eCO2 Emission Factor

eCO2 (MTCDE) = eCO2_r / 1000

CH4_r = Inventory * CH4 Emission Factor

CH4 (MTCDE) = CH4_r * CH4 GWP Value / 1000

N2O_r = Inventory * N2O Emission Factor

N2O (MTCDE) = N2O_r * N2O GWP Value / 1000

N2O (MT N) = N2O_r * N2O conversion factor (%)

NOx_r = inventory * NOx Emission Factor

NOx (MT N) = NOx_r * N2O conversion factor (%)

OtherN_r = Inventory * OtherN Emission Factor

Other N (MT N) = OtherN_r * N2O conversion factor (%)

**Calculations for refrigerants and chemicals for Carbon**

Refrigerant Chem (Refrigerant Chem (MTCDE)) = Inventory Value * Refrigerant Chem GWP Value / 1000

Footprint Calculations Part 1

**Carbon Footprint Calculation**

C Footprint (MTCDE) = CO2 (MTCDE) + eCO2 (MTCDE) + CH4 (MTCDE) + N2O (MTCDE) + Refrigerant Chem (MTCDE)

**Nitrogen Footprint Calculation**

N Footprint (MT N) = N2O (MT N) + NOx (MT N) + Other N (MT N)

**Phosphorus Footprint Calculation**

P Footprint (MT P) = Inventory Value (kg) * P Emission Factor / 1000

**Water Footprint Calculation**

If Category = Purchased Electricity:

W Footprint (gallons) = Inventory Value * Water Emission Factor * (10^(-12))*3600000/0.00378541178

If Category = Direct Water Use

W Footprint (gallons) = Inventory Value * Water Emission Factor * 1000000

If Category = All else:

W Footprint (gallons) = Inventory Value * Water Emission Factor

**Footprint Calculations**

Total Footprint Calculations

Total C Footprint = sum of all C Footprints by category (e.g., Agriculture Sources, Commuting, etc.)

Total N Footprint = sum of all N Footprints by category

Total P Footprint = sum of all P Footprints by category

Total W Footprint = sum of all W Footprints by category

**Cost Calculations**

C Cost Calculations

C Cost Min (MT) = 2 * C Footprint

C Cost Max (MT) = 50 * C Footprint

C Cost Mid (MT) = 30 * C Footprint

N Cost Calculations

N Fert Cost = 1000 * Baseline Fertilizer N (Other N)

N Fert NOx Cost = N Fertilizer Cost * 0.2

N Fert N2O Cost = N Fertilizer Cost * 0.03

N Fert NH3 Cost = N Fertilizer Cost * 0.13

N Fert SW Cost = N Fertilizer Cost * 0.21 (proportion that goes to SW (Houlton et al. 2013))

N Fert GW Cost = N Fertilizer Cost * 0.79 * 0.33 (proportion that goes to GW (Sabota et al. 2015))

N Fert Coast Cost = N Fertilizer SW Cost * 0.40 (proportion of SW that goes to coast (Sabota et al. 2015))

N_Cost_NOx_atm_h = 1000 * NOx (MTN) * 32.20 $/kgN Human respiratory health)

N_Cost_NH3_atm_h = 0 * 6.87 ($/kgN Human respiratory health (zero unless fertilizer - see below))

N_Cost_NOx_atm_e = 1000 * (NOx (MTN) * 0.43 # $/kgN visibility damages)

N_Cost_NH3_atm_e = 0 * 0.43 ($ / kgN visibility damages (zero unless fertilizer - see below))

N_Cost_N2O_atm_c = 1000 * N2O (MTN) * 15.07 ($ / kgN Greenhouse damages)

N_Cost_NOx_atm_c = 1000 * NOx (MTN) * (-5.02) ($ / kgN Climate Cooling Benefits)

N_Cost_NH3_atm_c = 0 * (-5.02) ($ / kgN Climate Cooling Benefits (zero unless fertilizer - see below))

N_Cost_NOx_ter_h = 1000 * NOx (MTN) * 0.12 ($ / kgN Damages to structures)

N_Cost_NH3_ter_h = 0 * 0.12 ($ / kgN Damages to structures (zero unless fertilizer - see below)

N_Cost_NOx_ter_a = 1000 * NOx (MTN) * 2.11 ($ / kgN Ozone damages to crops)

N_Cost_N2O_ter_a = 1000 * N2O (MTN) * 1.48 ($ / kgN UV damages to crops)

N_Cost_NOx_ter_e = 1000 * NOx (MTN) * 1.24 ($ / kgN Ozone damages to forests)

If sub category = Fertilizer

N_Cost_NOx_atm_h = N Fert NOx Cost * 32.20 # $/kgN Human respiratory health

N_Cost_NH3_atm_h = N Fert NH3 Cost * 6.87 ($ / kgN Human respiratory health)

N_Cost_NOx_atm_e = N_Fert_NOx Cost * 0.43 ($ / kgN visibility damages)

N_Cost_NH3_atm_e = N_Fert_NH3 Cost * 0.43 ($ / kgN visibility damages)

N_Cost_N2O_atm_c = N_Fert_N2O Cost * 15.07 ($ / kgN Greenhouse damages)

N_Cost_NOx_atm_c = N_Fert_NOx Cost * (-5.02) ($ / kgN Climate Cooling Benefits)

N_Cost_NH3_atm_c = N_Fert_NH3 Cost * (-5.02) ($ / kgN Climate Cooling Benefits)

N_Cost_NOx_ter_h = N_Fert_NOx Cost * 0.12 ($ / kgN Damages to structures)

N_Cost_NH3_ter_h = N_Fert_NH3 Cost * 0.12 ($ / kgN Damages to structures)

N_Cost_NOx_ter_a = N_Fert_NOx Cost * 2.11 ($ / kgN Ozone damages to crops)

N_Cost_N2O_ter_a = N_Fert_N2O Cost * 1.48 ($ / kgN UV damages to crops)

N_Cost_NOx_ter_e = N_Fert_NOx Cost * 1.24 ($ / kgN Ozone damages to forests)

N_SW_kg = 0.21 * 1000 * (NOx (MTN) + OtherN (MTN)) (all N sources (not N2O), proportion that goes to SW (Houlton et al. 2013))

N_GW_kg = 0.79 * .33 * 1000 * (NOx (MTN) + OtherN (MTN)) (proportion that goes to GW (Sabota et al. 2015))

N_coast_kg = 0.40 * N_SW_kg (proportion of SW that goes to coast (Sabota et al. 2015))

If Category is Fertilizer

N_SW_kg = N_Fert_SW Cost

N_GW_kg = N_Fert_GW Cost

N_coast_kg = N_Fert_coast Cost

N_Cost_aquaFresh_e =

N_SW_kg * 0.23 ($/kgN Lake waterfront property values) +

N_SW_kg * 0.19 ($/kgN Recreational Freshwater use) +

N_SW_kg * 0.01 ($/kgN Endangered species protection)

N_Cost_aquaGround_h =

N_GW_kg * 0.15 ($/kgN Replacement with bottled water) +

N_GW_kg * 0.60 ($/kgN Treatment of private well water)

N_Cost_aquaCoast_e =

N_coast_kg * 7.11 ($/kgN Recreational use of estuary)

N Costs Total

N_Cost_Total =N_Cost_NOx_atm_h + N_Cost_NH3_atm_h + N_Cost_NOx_atm_e + N_Cost_NH3_atm_e + N_Cost_N2O_atm_c + N_Cost_NOx_atm_c +

N_Cost_NH3_atm_c + N_Cost_NOx_ter_h +

N_Cost_NH3_ter_h + N_Cost_NOx_ter_a +

N_Cost_N2O_ter_a + N_Cost_NOx_ter_e +

N_Cost_aquaFresh_e + N_Cost_aquaGround_h +

N_Cost_aquaCoast_e

**References:**

Dodds, W.K., Bouska, W.W., Eitzmann, J.L., Pilger, T.J., Pitts, K.L., Riley, A.J., Schloesser, J.T. and Thornbrugh, D.J., 2009. Eutrophication of US freshwaters: analysis of potential economic damages*. American Chemical Society*. 43:12-19.

Compton, J.E., Harrison, J.A., Dennis, R.L., Greaver, T.L., Hill, B.H., Jordan, S.J., Walker, H. and Campbell, H.V., 2011. Ecosystem services altered by human changes in the nitrogen cycle: a new perspective for US decision making. *Ecology letters*, *14*(8), pp.804-815.

Kusiima, J.M. and Powers, S.E., 2010. Monetary value of the environmental and health externalities associated with production of ethanol from biomass feedstocks. *Energy Policy*, *38*(6), pp.2785-2796.
